# Candidate probiotic *Lactiplantibacillus plantarum* HNU082 rapidly and convergently evolves within human, mice, and zebrafish gut but differentially influences the resident microbiome

**DOI:** 10.1186/s40168-021-01102-0

**Published:** 2021-06-30

**Authors:** Shi Huang, Shuaiming Jiang, Dongxue Huo, Celeste Allaband, Mehrbod Estaki, Victor Cantu, Pedro Belda-Ferre, Yoshiki Vázquez-Baeza, Qiyun Zhu, Chenchen Ma, Congfa Li, Amir Zarrinpar, Yang-Yu Liu, Rob Knight, Jiachao Zhang

**Affiliations:** 1 School of Food Science and Engineering, Hainan University, Haikou, China; 2 UCSD Health Department of Pediatrics, University of California, San Diego, 9500 Gilman Drive, La Jolla, CA 92093 USA; 3 Center for Microbiome Innovation, Jacobs School of Engineering, University of California, San Diego, 9500 Gilman Drive, La Jolla, CA 92093 USA; 4 Biomedical Sciences Graduate Program, University of California, San Diego, 9500 Gilman Drive, La Jolla, CA 92093 USA; 5 Department of Bioengineering, University of California, San Diego, 9500 Gilman Drive, La Jolla, CA 92093 USA; 6 Key Laboratory of Food Nutrition and Functional Food of Hainan Province, Haikou, 570228 China; 7 UCSD Division of Gastroenterology, University of California, San Diego, 9500 Gilman Drive, La Jolla, CA 92093 USA; 8 VA San Diego Healthcare, 3350 La Jolla Village Dr, San Diego, CA 92161 USA; 9 Channing Division of Network Medicine, Department of Medicine, Brigham and Women’s Hospital and Harvard Medical School, Boston, MA 02115 USA; 10 Department of Computer Science and Engineering, University of California, San Diego, 9500 Gilman Drive, La Jolla, CA 92093 USA

**Keywords:** *Lactiplantibacillus plantarum*, Universal strategy, Adaptive evolution, Probiotic

## Abstract

**Background:**

Improving probiotic engraftment in the human gut requires a thorough understanding of the in vivo adaptive strategies of probiotics in diverse contexts. However, for most probiotic strains, these in vivo genetic processes are still poorly characterized. Here, we investigated the effects of gut selection pressures from human, mice, and zebrafish on the genetic stability of a candidate probiotic *Lactiplantibacillus plantarum* HNU082 (Lp082) as well as its ecological and evolutionary impacts on the indigenous gut microbiota using shotgun metagenomic sequencing in combination with isolate resequencing methods.

**Results:**

We combined both metagenomics and isolate whole genome sequencing approaches to systematically study the gut-adaptive evolution of probiotic *L*. *plantarum* and the ecological and evolutionary changes of resident gut microbiomes in response to probiotic ingestion in multiple host species. Independent of host model, Lp082 colonized and adapted to the gut by acquiring highly consistent single-nucleotide mutations, which primarily modulated carbohydrate utilization and acid tolerance. We cultivated the probiotic mutants and validated that these gut-adapted mutations were genetically stable for at least 3 months and improved their fitness in vitro. In turn, resident gut microbial strains, especially competing strains with Lp082 (e.g., *Bacteroides* spp. and *Bifidobacterium* spp.), actively responded to Lp082 engraftment by accumulating 10–70 times more evolutionary changes than usual. Human gut microbiota exhibited a higher ecological and genetic stability than that of mice.

**Conclusions:**

Collectively, our results suggest a highly convergent adaptation strategy of Lp082 across three different host environments. In contrast, the evolutionary changes within the resident gut microbes in response to Lp082 were more divergent and host-specific; however, these changes were not associated with any adverse outcomes. This work lays a theoretical foundation for leveraging animal models for ex vivo engineering of probiotics to improve engraftment outcomes in humans.

**Video abstract**

**Supplementary Information:**

The online version contains supplementary material available at 10.1186/s40168-021-01102-0.

## Background

Probiotics have been long used as gastrointestinal therapeutics under many health conditions [1]. Unlike other therapeutics, probiotics are live micro-organisms and typically leverage different strategies to adapt within the gut under high selective pressure [2, 3], e.g., spontaneous adaptive mutations [4, 5]. The gut-adaptive evolution of a probiotic genome can confer sufficient fitness advantages to engage in interactions with gut residents and host factors [6, 7], which eventually determine if and how long the probiotic can engraft for the intended therapeutic effect. The observed in vivo evolution of probiotics also presented novel opportunities to understand or leverage these gut-selective forces for genetic engineering of probiotics for better engraftment outcomes, such as *E*. *coli. Nissle* (EcN) [3]. However, for most probiotic strains, these in vivo genetic processes are still poorly characterized. The genomic contents of probiotics are highly distinctive and thus the corresponding adaptive behaviors should be characterized for a type strain at least within a species level. Furthermore, the probiotic adaptations and efficacy can be highly specific to its ecological niches, such as the configuration of indigenous gut microbiomes or host filtering forces [8, 9]. Therefore, more studies are urgently needed to comprehensively characterize the adaptive evolutionary behaviors of a probiotic strain under the diverse schemes. Among these, the host factor is often challenging to investigate but can provide important insight into improving the probiotic’s engraftment in the human gut [9, 10]. A wide array of animal models, including mice, flies, and zebrafish, have been used to elucidate the mechanism by which an engrafted probiotic such as *Lactiplantibacillus plantarum* (formerly known as *Lactobacillus plantarum*) adapts within the gut [11], and modulates host physiology [12]. However, the applicability of these findings from animal models to humans remains uncertain since each host species has a specific selection mechanism by which they regulate their resident microbial populations [13] and exogenous microbes entering the gut. Given that host factors strongly modulate the ecological niches such as the gut, the adaptive behaviors of probiotics in these environments are often host-dependent. Interestingly however, inter-species convergent adaptation of probiotics around specific metabolic functions may also occur [14]. Understanding the mechanism behind these adaptations may fundamentally update our knowledge of probiotic evolution and open venues for engineering desired traits of probiotics for humans by simulating evolutionary pressures such as passing them through the gut of an animal host or a similar artificial system. We can further ask if and how the fitness advantages of a probiotic gained from passing through the mouse gut can be stably transferred to a human host resulting in enhanced engraftment outcomes.

In addition to the adaptations of the probiotic within the gut, their presence can also induce strong ecological and evolutionary forces that could reshape the indigenous microbial communities. Many metagenomics studies explored the ecological impact of probiotic ingestion on gut microbiota, yet marginal changes in the composition of gut microbiota have been noted [10, 15–17]. But it is under-reported that the genetic composition of the human gut microbiome is constantly evolving under intrinsic forces such as aging, or external environmental disturbances such as diet [8, 18, 19] regardless of observable ecological changes. It is likely that evolutionary responses buffered a variety of environmental changes and further exerted a long-term effect on the population dynamics of evolving species [18, 20]. While probiotics were proposed to modulate the gut ecosystem for digestive health [3, 6, 21], they can also have a notable impact on the in vivo evolutionary trajectories or functions of those residents regardless of whether probiotics colonize or transiently pass through the gut [15]. However, no studies have systematically assessed how native gut microbiota adaptively evolve under the selection changes accompanying probiotics ingestion.

To address these questions, we employed *Lactiplantibacillus plantarum* HNU082 (Lp082), which was isolated from traditional fermented food [22] and whole-genome sequenced (PRJCA000348, PRJNA637783), as a model probiotic strain. It has gained increasing attention as its conventional characteristics of probiotic *L*. *plantarum* [12, 23] and specific functions such as hyperlipidemia prevention [24] and regulation of neurotransmitter secretion disorder. Meanwhile, we recently established the pipeline for efficiently isolating and identifying Lp082 (or its mutants) from fecal samples, which enables us to study its adaptive evolution *in vivo*. Here, we explored the effects of host-derived selection pressures (humans, mice, and zebrafish) on the genetic stability of probiotics and, in turn, its ecological and evolutionary impact on the indigenous gut microbiota using shotgun metagenomic sequencing and isolate resequencing methods.

## Results

Here, Lp082 was used as a model strain to compare the adaptive evolution patterns of a probiotic under different host selection pressures. For the mice probiotic group, 4 × 10^8^ CFU/g Lp082 was infused daily for 7 days. For the seven volunteers in the probiotic group, they were guided to consume vacuum freeze-drying powder including 7 × 10^9^ CFU Lp082 every day for 7 days. After stopping the probiotic consumption, we isolated the ingested Lp082 from fecal samples at different time points for further adaptive evolution analysis, while fecal samples were collected for metagenomic sequencing. Additionally, a zebrafish model was used to validate the convergence in the genetic variability of the ingested probiotic under distinct host selective pressures.

### The adaptive evolution of probiotics within the gut of distinct hosts

**Fig. 1:**
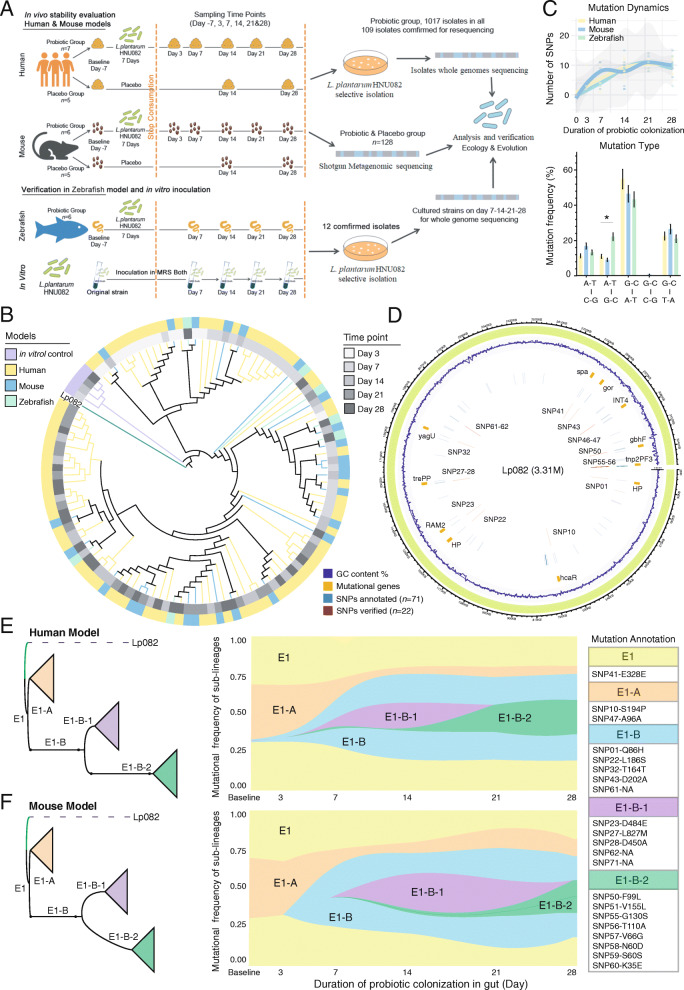
The in vivo adaptive evolutionary dynamics of probiotics over a 4-weeks sampling period in the gut of humans, mice and zebrafish. **A** The experimental design. We used Lp082 as a model probiotic strain to explore the effects of host-derived selection pressure (the mouse and human models were applied; the zebrafish model was used for further verification) on the genetic stability of the ingested probiotics and the impact of probiotic mutations on the indigenous gut microbiome of different hosts (the mouse and human model). First, we sequenced the complete genome of this model probiotic strain. We next isolated the probiotics from the feces of hosts at different time points to identify genetic mutations using whole-genome resequencing. Simultaneously, the original strain was continuously inoculated *in vitro* and sequenced to assess genetic mutation in the absence of host selective pressure. Next, we employed the metagenomic sequencing method to characterize the impact of probiotics ingestion on resident gut microbiota in humans and mice as compared to the placebo groups. **B** Phylogenetic tree constructed based on the SNPs of all Lp082 isolates. The different grey bars and color bars represent the strains isolated from different time points and different hosts, and the Lp082 was set as the root strain. The isolates were dominated by 22 SNPs during the probiotic colonization, especially in the first two time points of the human model. **C** The number of SNPs detected at every sampling time point (including the day 3, 7, 14, 21, and 28) in human, mouse, and zebrafish models (top panel). No significant difference in the number of SNPs at the end of the experiment was found among the three models. The mutation type of G-C to A-T was the most frequently detected in all three models, but the frequency of the mutation type of A-T to G-C was significantly higher in the zebrafish model than that in the other two models (bottom panel). **D** All confirmed SNPs and their gene locations are marked on the reference genome of Lp082. **E**, **F** (left panel) The simplified phylogenetic tree based on Lp082 isolates from the human (**E**) and mouse (**F**) model in all sampling time points. The tree is rooted in the ancient probiotic strain consumed and evolved into 3 groups (labeled in different colors) based on 21 SNPs in 5 branches. The evolutionary relationships of the 5 branches are visualized as E1, E1-A/B, and E1-B-1/2. **E**, **F** (right panel) The evolutionary dynamics of the 5 branches (in different colors) in the human and mouse model were constructed based on the mutation frequency of the 21 SNPs. A major difference in the evolutionary dynamics of branch E1-B-1 was observed between the human and mouse model, which consisted of the temporary evolutionary divergence in the second week of probiotic colonization

We employed the standard reference-based approach to explore the genetic changes of the consumed probiotics Lp082 under the in vivo natural selection in the gut of multiple hosts (humans, mice, and zebrafish) (Fig. 1A). We sequenced the complete genome of this reference probiotic strain, including one chromosome and four plasmids. Next, we isolated this strain from the feces of hosts at different time points for whole-genome sequencing. To identify and quantify the putative genetic mutations (such as single nucleotide variants) in the host-adapted strains, we compared the genome of all isolates with the reference genome. In total, 109 bacterial strains of Lp082 were isolated from feces or intestine content of three hosts, out of which 77 isolates were from humans, 25 from mice, and only 7 from zebrafish for the duration of the whole experiment (Fig. 1B, C, Table S1 and Table S2). A total of 71 putative single-nucleotide polymorphisms (SNPs) and 2 mobile genetic elements (Fig. S1B) were identified and annotated from genome sequencing data of in vivo-adapted probiotic strains in human, mouse, and zebrafish models, out of which only 22 SNPs could be experimentally verified using PCR (Fig. 1D). By contrast, under the in vitro condition, no SNP was annotated in Lp082 incubated in the de Man, Rogosa, and Sharpe (MRS) agar within 28 days (Fig. S1A), suggesting these adaptive mutations only occurred during the gut passage of this probiotic.

We next sought to characterize in vivo adaptive mutations of Lp082 across host models. First, despite the radically distinct host selection pressures, we did not identify any host-specific SNPs in the genome of Lp082. Furthermore, no between-host difference in the accumulated mutations, that is, average number of SNPs at the last time point was observed (human = 9.93; mouse = 10; zebrafish = 9; *p* = 0.926, Kruskal-Wallis test) as well as mutation types (Fig. 1C).

We next profiled and compared the temporal evolutionary dynamics of Lp082 over a 4-weeks sampling period between the humans and mice (Fig. 1E, F, Fig S1C-1G). As for all hosts, the mutational frequency of Lp082 steadily increased in the initial 2 weeks of gut colonization, suggesting the fitness advantages of lineages were carrying them relative to their ancestors. Since relatively few new SNPs occurred, the number of mutations tends to stabilize after the third week of colonization (Fig. 1C). Based on these adaptive mutations, we employed the phylogeny to identify five lineages of the candidate probiotic Lp082, which commonly emerged in humans and mice (Fig. 1E). We further sought to investigate how similar the evolutionary history of these co-existing probiotic lineages can be between two hosts (Fig. 1E, F). Overall, the temporal dynamics pattern of the probiotic lineages throughout the sampling period was highly conservative between humans and mice (*p* = 1e-4, *R* = 0.86, Mantel test based on Jaccard distance). Despite its occupation in the ecologically and spatially distinct niches, Lp082 acquired highly similar mutations in almost the same order and timing for the gut adaptation. At the initial stage of colonization, the sub-lineage E1 firstly emerged by acquiring a mutation SNP41 on the gene encoding IgG binding protein (Gene 2601). In the following sampling period, this lineage co-existed with other descendants with a consistently high frequency in the Lp082 population (> 50%) (Fig. 1E, F, Fig. S2A, Table 1), suggesting that Lp082 should persistently resist the host immunity for establishing and maintaining its ecological niche in the gut. Next, the lineages E1-A and E1-B appeared and maintained their proportion in the Lp082 population in the gut throughout the sampling period. The most notable adaptive mutations (SNP10, 47, and 32) from these lineages were involved in the genes (Gene1717, 0658, and 2804) encoding inner membrane protein response to acid pH, transcriptional activator for 3-phenylpropionic acid catabolism, and transpose, indicating Lp082 was actively developing the acid-tolerance capability in vivo by adaptive mutations. Around 7 days after probiotic ingestion, E1-B-1 emerged and carried the representative mutations (e.g., SNP 23) that can enhance the capability of rhamnose utilization (Gene 1257). Interestingly, this sub-lineage vanished at the end of our sampling period, and survived slightly longer in mice (day 28) than in human gut (day 21), which is the only observable difference in the probiotic evolutionary history in the gut between two hosts.

**Table 1:** The detailed information of the 22 SNPs

SNP ID	Location	Aa	Alt	Gene ID	MT	AAC	Gene name	Biological process	Protein annotation
SNP01	63020	C	A	Gene0056	N	Q86H	HP	NA	Transposase
SNP10	695080	T	C	Gene0658	N	S194P	hcaR	Transcription regulation	Transcriptional activator for 3-phenylpropionic acid catabolism
SNP22	1283071	A	G	Gene1205	N	L186S	HP	NA	Hypothetical protein
SNP23	1338981	A	C	Gene1257	N	D484E	ram2	Protein farnesylation	Bacterial alpha-L-rhamnosidase 6 hairpin glycosidase
SNP27	1589637	G	T	Gene1470	N	L827M	trePP	Carbohydrate metabolic process	Trehalose 6-phosphate phosphorylase
SNP28	1590767	T	G	Gene1470	N	D450A	trePP	Carbohydrate metabolic process	Trehalose 6-phosphate phosphorylase
SNP32	1861048	T	C	Gene1717	S	T164T	yagU	Response to acidic pH	Inner membrane protein response to acidic
SNP41	2799363	A	G	Gene2601	S	E328E	spa	Virulence	Immunoglobulin G-binding protein
SNP43	2854610	A	C	Gene2651	N	D202A	gor	Glutathione metabolic process	Glutathione reductase
SNP47	3007289	T	G	Gene2804	S	A96A	int4	DNA integration	Transposase
SNP46	3007122	T	G	Gene2804	N	S41A	int4	DNA integration	Transposase
SNP50	3212834	T	G	Gene3003	N	F99L	ybhF	Transport	ABC transporter ATP-binding protein
SNP51	3213000	G	T	Gene3003	N	V155L	ybhF	Transport	ABC transporter ATP-binding protein
SNP55	3310964	C	T	Gene3110	N	G130S	tnp2PF3	Transposition	Transposase
SNP56	3311024	T	C	Gene3110	N	T110A	tnp2PF3	Transposition	Transposase
SNP57	3311155	A	C	Gene3110	N	V66G	tnp2PF3	Transposition	Transposase
SNP58	3311174	T	C	Gene3110	N	N60D	tnp2PF3	Transposition	Transposase
SNP60	3311545	T	C	Gene3111	N	K35E	tnp2PF3	Transposition	Transposase
SNP59	3311468	G	A	Gene3111	S	S60S	tnp2PF3	Transposition	Transposase
SNP61	1976363	A	G	NA	NA	NA	NA	NA	NA
SNP62	1976367	G	T	NA	NA	NA	NA	NA	NA
SNP71	3271378	G	T	NA	NA	NA	NA	NA	NA

“Aa” represented the original single nucleotide; “Alt” represented the mutated single nucleotide; “MT” represented mutation type, synonymous mutations or non-synonymous mutations; “AAC” represented amino acid changes

**Fig. 2:**
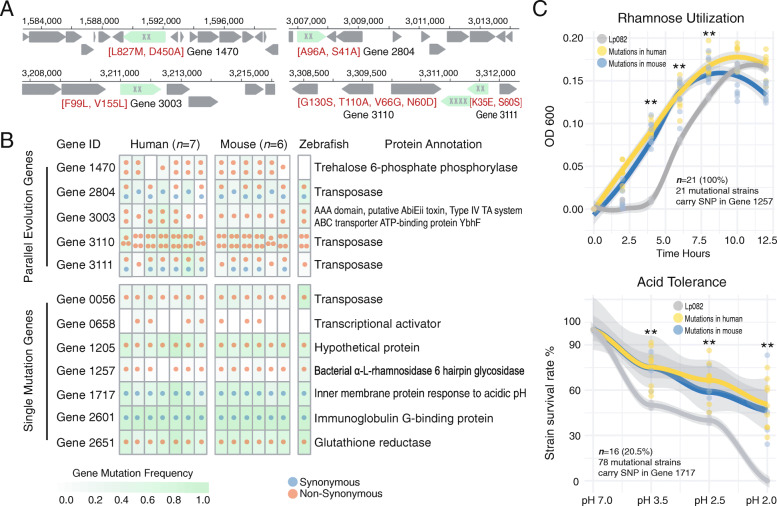
Genes that underwent in vivo evolution and the enhanced competitive fitness of the probiotic in mammalian hosts. **A** The locations of the five parallel evolutionary genes in the Lp082 chromosome. The genes highlighted by green color represent the parallel evolutionary genes and the “x” symbols represent the number of mutations detected in the gene. Annotations in red color represent the mutations that lead to functional changes in the amino acid sequence (i.e., L(Leu) to M(Met)). **B** Five probiotic genes underwent parallel evolution in different host species, while the other seven genes only have a single mutation. Each dot in the grid chart represents an independent mutation event and is also colored by types of mutation (synonymous or non-synonymous). The color within each cell represents the mutation frequency of a gene in all probiotic isolates from a host subject. **C** Either non-synonymous or synonymous SNPs can confer the growth benefits of probiotics in the host gut. The phenotypic verification experiments of probiotic isolates related to the rhamnose utilization (top panel) and acid tolerance (bottom panel). A non-synonymous SNP identified in Gene 1257 (annotated as bacterial alpha-L-rhamnosidase 6 hairpin glycosidase) from 21 mutational isolates (100%). This SNP conferred growth benefits to all isolates by utilizing the rhamnose more rapidly than the original strain. Remarkably, a synonymous SNP in Gene 1717 (annotated as inner membrane protein response to acidic) was found in 78 isolates. Among these, 16 mutational isolates (20.5%) improved the performance of this strain in acid tolerance regardless of a synonymous SNP

Next, we set to determine whether any genes in the probiotic genome had a similar pattern of adaptive mutations across independent lineages or host subjects. We focused on genes that had at least two SNPs and a maximum distance of 2000 bp between the genomes of the independent lineages and distinct host subjects or host species, indicative of parallel evolution. Such adaptive mutations identified from a gene are not likely a consequence of the genetic drifts, indicating that these genes are under natural selection. Among all 12 mutated genes, we identified five such genes from all 109 isolates encoding trehalose 6-phosphate phosphorylase, transposase, and ABC transporter ATP-binding protein (Fig. 2A). Remarkably, these genes carried consistent multiple mutations across most host subjects of humans, mice and zebrafish (Fig. 2A, Table S3). Next, we identified the other seven genes as single-mutation genes involved in bacterial alpha-l-rhamnosidase 6 hairpin glycosidase, immunoglobulin G-binding protein, glutathione reductase, inner membrane protein response to acidic pH, and transcriptional activator for 3-phenylpropionic acid catabolism (Fig. 2B). Collectively, the presence/absence patterns of adaptive mutations in each of 12 genes are highly consistent within and between host species (Fig. 2B), strongly demonstrating a universal adaptive strategy of this probiotic strain under varying selection pressures.

We next tested if those SNPs conferred the fitness advantage of Lp082 in the host gut using an in vitro model. We selectively validated the phenotypic changes of the host-adapted isolates related to the rhamnose utilization and acid tolerance (Fig. 2C, Fig. S2B and S2C). We focused on a non-synonymous SNP in Gene 1257 (annotated as bacterial alpha-l-rhamnosidase 6 hairpin glycosidase), which was identified in 21 host-adapted isolates. Intriguingly, all these mutants exhibited a higher capability in utilizing rhamnose in vitro than the original strain. While those mutants rapidly responded to the rhamnose substrate in the medium, the reference strain did not grow in the early 3 h after its inoculation. Furthermore, mutational isolates from humans and mice exhibited a highly consistent growth pattern. The human-derived strains just have a slightly longer period of exponential phase than did mouse-derived strains. Next, we tested the acid tolerance of 78 mutational isolates with a synonymous SNP in Gene 1717 (encoding inner membrane protein response to acidic pH) independently. Among those 78 mutants, 16 (20.5%) exhibited a significant higher survival rate under each of low pH conditions (*p* < 0.05, pH = 3.5, 2.5, 2.0 respectively) than the original strain. Notably, the mutant isolates also remained viable following 9 month of storage in − 80 °C.

### Compositional and functional alterations of resident gut microbiota in response to probiotic engraftment

**Fig. 3:**
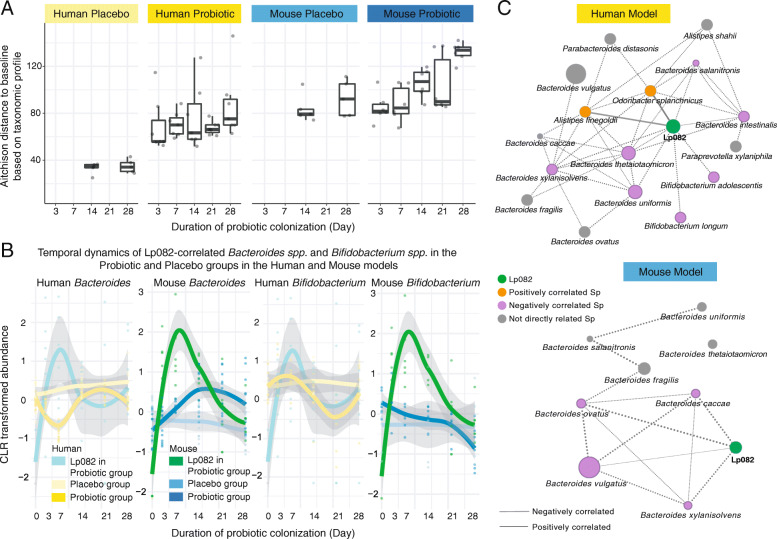
The microbiome response in the gut to the probiotic engraftment in humans and mice. **A** The Aitchison distance between the samples in baseline and other time points both in the human and mouse model. The colored points represented the samples at different time points. We found that interindividual heterogeneity in the human gut microbiota is greatest among those in the gut microbiota of all host models. **B** The temporal dynamic of 19 intestinal species belonged to the genera *Bacteroides* and *Bifidobacterium*, which were commonly identified in both human and mouse models. The dynamics of the same species residing in both humans and mice were highly divergent, suggesting niche-specific adaptation strategies of resident gut microbes responding to probiotic invasion. **C** The ecological relationships between the Lp082 and resident intestinal microbes are visualized by the co-occurrence networks in the human and mouse model. The nodes in different colors respectively represent Lp082 (green), the probiotic positively correlated species (orange), the probiotic negatively correlated species (purple), and the species indirectly correlated with the probiotic (grey). The correlation strength between nodes (species) was calculated by the SpiecEasi based on the CLR-transformed microbial relative abundance. The thickness of an edge represents the correlation strength between two nodes. A dashed edge indicates a negative correlation while a solid edge indicates a positive correlation between microbial species

We next examined how the resident gut microbiota ecologically responded to probiotic engraftment. Compared to the placebo group, the ingestion of Lp082 introduced a notable transient fluctuation in the taxonomic structure of indigenous microbial communities in both humans and mice (Fig. 3A, Fig. S3A and Fig. S3B). In humans, the probiotic intake did not significantly alter the overall taxonomic and functional composition of gut microbiome at any time points compared to baseline (*R*^2^ = 0.088, *p* = 0.979; PERMANOVA, Fig. 3A). This is consistent with previous observations [10, 16, 17]. The absence of large probiotic-derived influences on the host microbial composition while desirable, may result from a high level of individual variation or resilience in the human gut microbiota prior to ingestion. By contrast, we observed a strong impact of probiotics on the structure and function of the more homogenous mouse gut microbiota (*R*^2^ = 0.28, *p* = 0.005; PERMANOVA, Fig. 3A, S3C, S3D). Compared to the baseline, the structure of the mouse gut microbiome significantly changed on days 7, 14, and 28 after probiotic ingestion (*R*^2^ = 0.28, *p* = 0.005 on day 14; *R*^2^ = 0.19, *p* = 0.039 at day 21; *R*^2^ = 0.59, *p* = 0.004 at day 28; PERMANOVA). These observations were confirmed with both Aitchison (Fig. 3) and Bray-Curtis (dis)similarity distances (Fig. S3A and Fig. S3B).

Next, we sought to identify metagenomic markers associated with probiotic intake. Specifically, we defined a metagenomic marker according to the following conditions: (1) it significantly changed over time (e.g., from day 0 to 14) in the probiotic group; (2) it maintains stable abundance during the experiment in the placebo group; (3) it was significantly different in abundance at a given time point (such day 14) between the two groups. In the human population, no organismal or functional markers were identified based on these criteria which may be due to large inter-individual variations in the gut microbiome. In the mouse model, *Faecalibaculum rodentium* and 10 species from *Bacteroides*, including *B*. *caccae*, *B*. *ovatus*, *B*. *vulgatus*, *B*. *xylanisolvens*, etc., increased significantly in response to probiotic intake. Another 12 species including *Prevotella dentalis*, *P*. *oris*, and *Pseudomonas fluorescens* decreased sharply (Fig. S3C). Meanwhile, we also identified a total of 21 metabolic pathways, including d-galacturonate degradation, pyruvate fermentation to acetate and lactate, starch degradation, tetrapyrrole biosynthesis, l-citrulline biosynthesis, glycogen biosynthesis, and l-isoleucine biosynthesis, to be enriched during probiotic colonization (Fig. S3D).

In order to compare microbiome responses to probiotic colonization across host species, we here focused on 19 microbial species commonly found in both mammalian hosts, which taxonomically belong to the genus of *Bacteroides* or *Bifidobacterium* (Fig. 3C)*.* We found that these 19 microbial species residing the human and mouse gut had distinct temporal dynamics in response to probiotics ingestion. This suggests that the ecological response of resident microbes to probiotic engraftment highly depends on the host or gut microbiota context (Fig. 3B).

### Co-occurrence relationship between Lp082 and resident microbes in the gut of humans and mice

We next constructed a co-occurrence network in each host species to identify the correlation between resident microbes and the ingested probiotic. The co-occurrence networks were constructed using SpiecEasi [25] based on the species-level taxonomic profiles derived from shotgun metagenomic sequencing data (Fig. 3C). In the human model, we identified 10 species whose abundances strongly correlated with that of Lp082: *Alistipes finegoldii* and *Odoribacter splanchnicus* positively correlated with Lp082, while several *Bacteroides* spp. and *Bifidobacterium* spp. negatively correlated with Lp082. In the mouse model, we identified 4 *Bacteroides* species that were strongly negatively correlated with Lp082, these were *B*. *caccae*, *B*. *vulgatus*, *B*. *ovatus*, and *B*. *xylanisolvens*. We found that no common resident gut microbes across host species showed consistent correlation with Lp082. Except for *B*. *xylanisolvens*, there were no common negatively correlated bacterial species found between the human and mouse models (Fig. 3C). With the exception of *B*. *xylanisolvens*, most microbial competitors of Lp082 in the mouse model were also identified in the human gut; however, none of them exhibited the same co-occurrence relationship in the network analysis.

### Profound evolutionary changes within the resident gut microbiota in response to probiotic engraftment

**Fig. 4:**
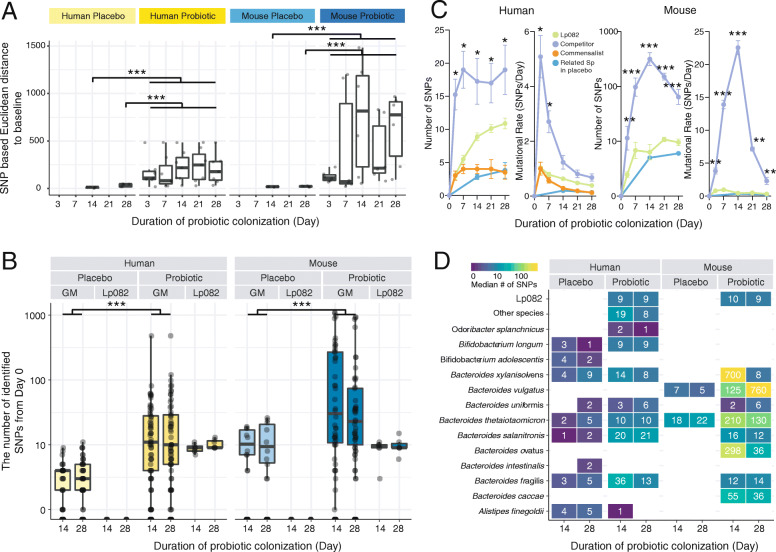
The rapid co-evolution of the ingested probiotic and resident gut microbiota of humans and mice within 28 days. **A** The Euclidean distance based on the number of SNPs identified from day 0 to other time points during the probiotic colonization in human and mouse models. It strongly indicated an intensive evolutionary response in resident microbiota due to probiotic intake. **B** The distribution of the mutations identified in the candidate probiotic Lp082 and resident gut microbiota from the probiotic and placebo group in both human and mouse models. Each dot in the boxplot represents the number of SNPs that occurred on a microbial strain in the gut of a host subject as compared to that on day 0. The “GM” is the abbreviation of “gut microbiota”. **C** The ecological relationship with Lp082 determined the number of SNPs that occurred on a resident microbial strain. Overall, the number of SNPs of probiotic “competitors” (orange, such as *Bacteroides spp.* and *Bifidobacterium spp.*) was significantly greater than that of “non-competitors” of this probiotic (orchid) or this probiotics (green) in the human model. In the mouse model, the adaptive mutations occurring in those probiotic competitors were one to two orders of magnitude more than those identified in the probiotics over the 28-day sampling period. The least mutations were identified in the placebo group (blue line). **D** The heat map indicates the median number of SNPs identified in each microbial species at day 14 and day 28 in each host group compared to day 0. Asterisks: statistical significance (*p < 0.05, **p < 0.01, ***p < 0.001)

Probiotic intake can give rise to dramatic changes in the genetic compositions in the resident species, yet it was surprisingly underreported. Here, we studied the within-genome evolution of approximately 37 prevalent microbial strains in the human or mouse gut. We observed a striking difference in the overall number of adaptive mutations in the resident microbiota between placebo and probiotics groups (Fig. 4A). The average number of mutations that occurred in the placebo group is only 1.92 and 1.69 for humans and mice, respectively. By contrast, probiotic intake gave rise to a dramatic change in mutation frequency in the resident gut microbes, which reached up to 16.90 or 78.02 per species in the human and mouse model, respectively (Fig. 4B, Fig. S4A). This indicated that a resident strain can accumulate on average 4.33 or 7.67 SNPs per day in humans or mice, respectively, which was remarkable given the high inter-individual variation in the gut microbiota of both humans and mice. Conversely, the genome of Lp082 was relatively stable: only 8 and 10 SNPs were detected on days 14 and 28 respectively (Fig. 4B). We next asked if these adaptive behaviors of residents might give rise to compositional or functional changes in the communities. In the mouse gut, the more mutations occurred in resident gut microbiota, the more pronounced shifts can be observed in the taxonomic or functional profiles after probiotic ingestion (Mantel test; Fig. S4B). This suggested that adaptive mutations could be a strong driving force reshaping the resident gut microbiota.

We next sought to characterize the distribution of these adaptive mutations among resident microbial strains. We found that the majority of mutations distributed in *Bacteroides* spp. and *Bifidobacterium* spp., which were mostly identified as ecological competitors of Lp082 in the resident gut microbiota of either humans or mice. We further demonstrated that the putative ecological competitors (such as *Bacteroides* spp. and *Bifidobacterium* spp.) proactively responded to dietary probiotics by accumulating significantly more adaptive mutations than non-competitors of this probiotic (*p* < 0.05, Wilcoxon rank-sum test, Fig. 4C, S4A). Remarkably, in the mouse model, mutations occurred in probiotic competitors were one to two orders of magnitude greater than those in the related species in the placebo group or the probiotic group over 28 days. Therefore, we provided the clue about the potentially causal links between evolutionary and population dynamics in the resident gut microbiota.

We next characterized how evolutionary dynamics in resident gut microbiota differed between humans and mice. We observed that the resident microbes in the mouse gut accumulated many more adaptive mutations than those in the human gut (Fig. 4C, D, Fig. S4C). For example, *B*. *xylanisolvens* is one of the overlapped members in the gut microbiota of both humans and mice, being negatively correlated with Lp082 in both hosts. After 14 days of probiotic ingestion, we identified on average 596 within-host SNPs in the mouse gut microbiotas, whereas only 15 SNPs identified on average for a human individual in the gut microbiota (Fig. 4D). These may suggest probiotics intake imposes more intensive selection pressure to resident gut microbiota in the mouse than does in the human. Next, we observed a substantial functional difference in the adaptive-mutated genes of the resident gut microbiota between human and mouse models. The human-associated mutations primarily occurred in the genes related to cAMP-binding proteins and conjugative transposon protein, whereas mouse-associated mutations involved in more diverse functions, such as the degradation and utilization of the complex carbohydrates (beta-galactosidase, phosphoglycolate phosphatase, 3-oxoacyl-[acyl-carrier protein] reductase, and galactoside O-acetyltransferase), Tetracycline resistance element mobilization regulatory protein, Asparaginyl-tRNA synthetase, DNA primase, conjugative transposon protein, and Transposase. There was only one gene (traG) that was consistently mutated in both the human and the mouse models, which related to the conjugative transfer of plasmid RP4 (TraG-like proteins are essential components of type IV secretion systems [26]). Collectively, even though the engrafted probiotic applied universal strategy in the distinct hosts, the indigenous microbiome response to its colonization was highly divergent, underscoring the importance of careful interpretation of ecological and evolutionary patterns identified in the mouse model for human-related translational studies.

## Discussion

Probiotics are live microorganisms that, in sufficient dose, confer a benefit to the host, and are widely used for improving digestive health [1]. However, their genomes and functional traits can vary during administration due to the myriad selection pressures in the host luminal environment [27]. The gastrointestinal tract harbors a large collection of microorganisms. The resident gut microbes are typically well adapted to their host, including the immune system and fluctuations in environmental resources over a relatively long period, resulting in higher competitive fitness over any potential invaders [18]. Changes in environmental selection pressures, such as competitions for limited carbon sources, force new microbes to dramatically reshape their genome and functional characteristics in a short-time scale.

In the present study, we demonstrated how the candidate probiotic Lp082 can evolve within the human and mouse gut by acquiring heritable changes to its genome. We found that Lp082 accumulated single-nucleotide mutations that modulate rhamnose utilization and acid tolerance in two mammalian host models. The significant improvement in rhamnose utilization can be attributed to a nonsynonymous mutation in the gene related to bacterial alpha-l-rhamnosidase 6 hairpin glycosidase. Furthermore, the findings also indicated that rhamnose can serve as a strain-specific prebiotic [28] that enhances the survival of Lp082 in the host gut. More interestingly, a synonymous mutation in the gene related to the function of inner membrane protein might respond to acidic pH. 20.5% (*N* = 16) of 78 mutational strains with this SNP had better performance in acid tolerance. Similarly, Crook et al. reported that probiotic EcN gained in vivo fitness by accumulating mutations related to carbohydrate utilization and acid tolerance within the mouse gut microbiome [3]. As a Gram-negative bacterial probiotic, EcN acquired 456 (336 unique) evolutionary changes across 171 genes within 35 days, while Lp082, a Gram-positive bacterial probiotic, only accumulated 22 SNPs across 12 genes within 28 days. This may suggest higher genome flexibility or instability of a gram-negative probiotic over gram-positive ones (such as conventional lactic acid bacteria) under in vivo selection.

Knowledge of normal genomic variations within and between host species can further allow us to develop new probiotics or modify existing strains that can engraft and colonize a human host, adding desired functions, safely, effectively, and reliably [6, 7]. For example, it is translational promising if probiotics can be genetically engineered by means of experimental evolution in the animal hosts where in vivo fitness can be improved under the human gut selection pressure [3]. While some animal models including mice and flies have been employed to study adaptive evolution of probiotics [11, 12], no studies have applied animal-gut-adapted probiotics to humans. Firstly, conducting in-deep study of in vivo adaptive evolutionary behaviors of individual probiotic strains is challenging as probiotic characteristics are highly strain-specific and the in vivo evolutionary behaviors can be more divergent. Next, the in vivo adaptation can also be shaped by multiple intrinsic and extrinsic factors and thus highly unpredictable. A comprehensive systematic investigation would be required to address the confounding of probiotic strains: host microbiome diversity, diet, and various other host factors. Crook et al. compared the genome adaptation of EcN in the host gut with different microbiome complexities [3]. They showed that as a gut commensal, this strain accumulated fewer mutations in the “high diversity” gut microbiome, while its genome varied more in the “low diversity” microbiome. By contrast, we found that Lp082 maintained a much higher genetic stability than EcN regardless of the stark differences in the microbiome complexity between human and mouse gut. Other than that, its adaptive evolutionary changes were not differentiated by the host factors from humans and mice. We argue that the universal adaptive strategy of Lp082 against selection pressures from multiple hosts could suggest the transferability of the fitness advantages of this probiotic from mice to humans, which definitely merits further investigation.

Probiotics adapt and evolve in vivo in order to survive. They also influence selection pressures on resident microbial strains. Here, we systematically investigated the ecological and evolutionary impacts of a probiotic *L*. *plantarum* on the resident gut microbiome in humans and mice. First, we observed a minimal ecological change including compositional or functional changes in the human gut due to probiotic ingestion which is consistent with previous studies [16, 29]. By contrast, the resident population of gut bacteria can rapidly and extensively evolve within 3–7 days after probiotic administration in both humans and mice. Most within-genome changes persisted for 28 days in our experiment. In the placebo group, very few evolutionary changes in resident microbes were found over time. Notably, strains competing with Lp082 account for the largest proportion (human: 74.36%; mouse: 77.68%) of evolutionary events in the whole resident gut microbial community. As described by the “Red Queen hypothesis” [6], microbial competitions within our gut ecosystems can force the acceleration of microbial evolution possibly without any observable ecological changes, resulting in apparent stability. It also suggested that the common or even daily supplement of probiotics or fermentation foods could alter the evolution and ecology in the resident gut microbiome, which was largely overlooked. Given the substantial number of adaptive mutations accumulated, it is still unknown if they can potentially alter the ecological/metabolic functions of gut microbiota in the long-term run. Therefore, the ecological and evolutionary impact of dietary probiotics on gut microbiota should be taken into account prior to administration [30]. Accordingly, future work should carefully reflect the limitations in the present evaluation system of probiotic efficacy in order to better design a framework for systematic assessment of interactions between probiotics, gut microbiome, and host over time.

## Conclusions

Collectively, our results demonstrated that *L*. *plantarum* can apply a highly convergent adaptation strategy in diverse host environments. Our findings shed new light on how *L*. *plantarum* adapts within different gut environments and lays the foundation for leveraging animal models for ex vivo engineering for enhanced probiotics engraftment in humans. Although this approach is promising, there are several factors that could hinder between-species translatability. Namely, engraftment is likely a microbiome-mediated feature and the high variability between human populations [31], or even the personal variability within a subject’s microbiome [32] could result in different engraftment events. On the other hand, as a means of modulating gut microbiome for human health, probiotic ingestion can lead to complex and widespread evolution in the gut microbiome that was often overlooked, underscoring the importance of systematic assessment of probiotics use especially from the evolutionary point of view.

## Methods and materials

### The experimental design

In this study, we used Lp082 as a model probiotic strain to explore the effects of host-derived selection pressure (the mouse and human models were applied, the zebrafish model was used for further verification of adaptive mutations) on the genetic stability of the consumed probiotics and the impact of probiotic mutations on the indigenous gut microbiome of different hosts (the mouse and human model). First, we sequenced the complete genome of this model probiotic strain, including one chromosome and four plasmids. Secondly, we isolated the probiotics from the feces of hosts at different time points to identify genetic mutations using whole-genome resequencing. Simultaneously, the original strain was continuously inoculated in vitro and sequenced to assess genetic mutation in the absence of host selective pressure. Next, we employed the metagenomic sequencing method to characterize temporal dynamics of the abundance of probiotics strain and other gut-microbiota members and genetic variations in the community level after probiotic ingestion and confirming the impacts by comparing the results with that in the placebo group. Last but not least, the difference in genetic variations of Lp082 and its impact on the indigenous gut microbiota among hosts were highlighted in this study.

In the mice (C57BL/6, 5 weeks age) experiments, each animal was housed in a single cage. Room temperature was 26 °C and the padding was replaced once every day. All mice were divided into two groups, the probiotic group (*n* = 6), and the placebo group (*n* = 5). For the mice in the probiotic group, Lp082 (about 4 × 10^8^ CFU/g with the fodder) was infused daily for 7 days. Fresh feces were collected from each cage for bacterial isolation on days 3, 7, 14, 21, and 28 after stopping probiotic feeding. Three pieces of fresh excrement were placed in a 7 mL sample tube, 4.5 mL saline (0.85%) was added after sterilization and then homogenized with homogenizer. Then the fecal samples were diluted and coated for Lp082 isolation as well as for shotgun metagenomic sequencing. For the mice in the placebo group, the same feeding method was performed only without the probiotic infused. The fresh feces were collected from each cage on days 0, 14, and 28 for shotgun metagenomic sequencing.

For human participants, each individual was informed of the experimental guidelines and details and consent obtained; 12 volunteers agreed to participate in the experiment. They were randomly divided into 2 groups including the probiotic group (*n* = 7, 4 females) and the placebo group (*n* = 5, 2 females). During the experiment, the subjects were asked to avoid ingesting any probiotic product or antibiotic and to maintain their regular diet. All healthy participants finished the whole experiment including 6 females and 6 males aged from 18 to 20 (BMI 19.19–22.49), they did not have inflammatory bowel disease or diabetes and had not used antibiotics for at least 3 months prior to sampling. For the seven volunteers in the probiotic group, they were asked to consume vacuum freeze-drying Lp082 powder (including 7 × 10^9^ CFU live strains) 2 g every day for 7 days [33], and fecal samples were collected on days 3, 7, 14, 21, and 28 after stopping probiotic consumption. Fresh feces were placed in a sterile fecal sampling tube, then the diluted feces samples spread to the MRS agar plate for Lp082 isolation as well as for shotgun metagenomic sequencing. In the placebo group, the five volunteers were requested to maintain a regular diet during the whole experiment, and their fresh feces were collected on days 0, 14, and 28 for shotgun metagenomic sequencing. The study was reviewed and approved by the Ethics Committee of Hainan University (HNU-2018037, Haikou, China), and informed consent was obtained from all volunteers before they enrolled in the study. The participants provided written informed consent to participate in the study. Sampling and all described subsequent steps were conducted in accordance with the approved guidelines.

Additionally, a zebrafish model was used to validate our findings related to the impacts of host intestinal selective pressure on the genetic stability of the ingested probiotic. A total of 20 fish (15 weeks age) tanks (15–18 zebrafish in each tank) were used in this experiment. The water was changed and the fish were fed at 9:00 am daily; the capacity of feed was calculated according to 3% of the body weight of each fish per day. After the adaptation period, *Lp082* of 10^8^ CFU/g was fed for 7 days with the fodder [23]. The intestines of 5 fish were taken and homogenized with 1 mL saline (0.85%) in 1.5 mL EP tube and in 3 days, 7 days, 14 days, 21 days, and 28 days after stopping probiotic feeding. Then the intestine samples were diluted and coated for *Lp082* isolation.

### Isolation and confirmation of the ingested probiotic strain (Lp082) in the feces

The reference strain Lp082 used in the present study was first isolated in traditional fermented food in Hainan province of China [22] and further certified as a probiotic due to the common characteristics of probiotic *Lactobacillus* spp. and specific functions such as hyperlipidemia prevention [24] and neurotransmitter secretion disorder regulation. We obtained the complete genome of the strain with functional annotations in our previous research (PRJCA000348, PRJNA637783). By comparing with other genome sequenced *L. plantarum* strains (Table S4), we identified strain-specific primers of Lp082, which was used for genetic confirmation of isolates in the next step. The detailed experimental steps for Lp082 isolation from feces and confirmation were as follows:

1. Feces/intestinal contents were taken from the sample tube, and sterilized saline (0.85%) was added to the sample tube. After stirring with a homogenizer, the samples were diluted and coated. The mixture of 0.5 mL and 4.5 mL of 0.85% saline (NaCl) was recorded as 10-1, 10-2, 10-3, 10-4, 10-5, 10-6 were obtained.
2. Each diluted gradient was selected for coating. About 100 μL mixed liquid was taken on solid de Man, Rogosa and Sharpe (MRS) medium and 50 μL 1280 μg/mL vancomycin solution and 50 μL 1280 μg/mL norfloxacin solution was added evenly. Date, number, weeks, and concentration were marked on a petri dish, which was placed in an incubator at 37 °C for 48 h.
3. The single colony cultured in the medium was selected and again cultured in the test tube of 5 mL MRS broth medium. After being shaken uniformly, the bacteria were cultured in an incubator at 37 °C for 48 h.
4. Colony PCR was used to validate the bacterial solution by strain-specific primers. Samples that could be amplified were preserved and whole-genome sequenced for further validation.

### Isolate whole genome sequencing and data quality control

Bacterial genomic DNA was extracted from the isolates for whole-genome sequencing. Using high-throughput sequencing, paired-end reads (2 × 150 bp) library of each single bacteria sample prepared on the Illumina Hiseq 2500 platform in Shanghai Personal Biotechnology company (Shanghai, China). Quality control was carried out with FastQC, AdapterRemoval (v2.1.7) was used to remove the joint contamination [34], and SOAPec (v2.0) software was used to carry out quality correction based on Kmer frequency [35].

### SNP calling and construction of the phylogenetic tree of mutants

To estimate the evolutionary distance between isolates across the 3 models and phylogenetic tree construction, we aligned all short reads to the reference genome of Lp082 for SNPs identification. Reads were aligned using Bowtie2 [36] (with alignment parameters: bowtie2 -p -x --no-mixed --very-sensitive --n-ceil 0,0.01 -1 -2 | SAMtools sort -O bam -@ 24 -o - > *.bam). Candidate SNPs were identified and filtered with SAMtools [37]. In particular, candidate SNP positions were identified if at least one pair of isolates were discordant on the called base and both members of the pair had: FQ scores (produced by Bcftools) less than 60, at least 7 reads that aligned to both forward strands and reverse strands and a major allele frequency of at least 90%. If the median coverage across samples at a candidate position was less than 10 reads or if 33% or more of the isolates failed to meet the filters described above, this position was discarded. We generated a neighbor-joining tree from the concatenated list of variable positions from conserved genomic regions present in all isolates from all samples by MEGA-X and iTOL software [38]. When computing the distance between each pair of isolates, we only used variable positions that had unambiguous nucleotide calls from both isolates.

### Calculating the distance to the most recent common ancestor (dMRCA) and the mutation type

To calculate dMRCA for each model at each time point, we counted the number of positions at which the called allele was different from the ancestral allele for each isolate, assessing only SNP positions that were polymorphic among isolates from the particular time point, and averaged the results. SNPs were categorized into 6 types, based on the chemical nature of the single nucleotide changes. We computed the mutation spectrum for each model and then computed the mean and standard deviation of each of the 6 types. The frequency of G-C to A-T mutation was the most abundant in all three models. The frequency of A-T to G-C mutation was significantly higher in the zebrafish model than that in the other two models.

### Identification of the mobile elements of Lp082 during ingestion

To identify mobile elements of the strain Lp082, we performed the pan-genome analysis of all strains (*n* = 109) isolated in this study, including the original reference strain and all isolates from different hosts and time points. In general, the whole genomic sequencing reads of each isolate was assembled by SPAdes with the parameters as below: spades.py -t 24 -k 21,33,55,77,89 --careful --only-assembler [39]. To delineate the core genome of all strains, we mapped the assembled contigs (including only those with the length of > 500 bp) of each strain against the reference strain of Lp082 using BLASTn [40] with the identity level ≥ 90% and an *e* value < 1e-5. Then, we obtained a set of strain-specific contigs by excluding the contigs that were mapped to the core-genome. Further, to remove the potential redundancy in the strain-specific sequences, we compared each pair of these sequences using BLAT [41] with an identity level ≥ 90% and an alignment length ≥ 85%, which resulted in a set of non-redundant contigs specific to strains that can be also termed as the accessory genome. We reason that all the contigs in the accessory genome of this strain can be inferred as the “mobile elements” inserted into the reference genome (Lp082) during microbial colonization.

To investigate the presence/absence of each potential mobile element in a given isolate, we calculated the read coverage and depth of each isolate against the accessory genome: coverage > 80% and depth > 60× were considered to be present, while coverage < 20% or depth < 60× were regarded as absent [42]. Accordingly, we calculated the coverage and depth of each mobile element in each isolate (Table S5).

### Identification of the genes with parallel evolution among the models

We counted a gene as under parallel evolution if, in at least one host, the gene had multiple independent SNPs and more than 1 SNP per 2000 bp (to account for the fact that long genes are more likely to be mutated multiple times by chance). Cases in which two SNPs in the same gene that always occurred together in the same isolates were not included as parallel evolution [8]. Based on these principles, a total of 5 genes related to carbohydrate utilization and transposase were identified under parallel evolution.

### Evolutionary dynamics of the strain Lp082 in the gut

While too few probiotic isolates (*n* = 7) in the zebrafish model over the 28-day experiment, we only characterized the evolutionary dynamics of the probiotic strain within the human and mouse model. To increase the reproducibility of SNPs studied, we only focused on SNPs that should be identified from at least 4 isolates. For each of the 22 SNPs that met this criterion, we calculated the frequency of reads at each SNP position that agreed with the mutation (derived) allele. To fill the time points where no strain was isolated, we generated a continuous relative abundance of SNPs over time by continuous bezier interpolation [43]. To confirm the parent and child relationship of these SNPs, the phylogenetic trees were constructed for all strains isolated in the human and mouse model. To visualize parent and child lineages separately, we subtracted the relative abundance of a parent sublineage by the sum of relative abundances of its child sublineages. When the combined relative abundance of child sublineages exceeded that of their parent sublineage, we set the frequency of the parent sublineage to 0. At last, the Muller plot was employed for dynamic visualization [43]. We did not build the model of the evolutionary dynamics of this probiotic in the zebrafish model, as too few isolates (only seven) were found.

### Metagenomic DNA extraction and shotgun metagenomic sequencing and data quality control

The QIAamp® DNA Stool Mini Kit (QIAGEN, Hilden, Germany) was used for DNA extraction from the fecal samples. The quality of the extracted DNA was assessed by 0.8% agarose gel electrophoresis, and the OD 260/280 was measured by spectrophotometry. All of the DNA samples were subjected to shotgun metagenomic sequencing by using an Illumina HiSeq 2500 instrument in the Novogene Company (Beijing, China). Libraries were prepared with the paired-end reads (2 × 150 bp). The raw reads were trimmed using Sickle (https://github.com/najoshi/sickle) and subsequently aligned to the human genome to remove the host DNA fragments.

### Identification of metagenomic species, microbial functional genes, and metabolic pathway annotation

Bracken [44] was applied for metagenomic species identification and abundance estimation (Table S6). For metagenomic functional features and metabolic pathway annotation (Table S7), HUMAnN2 was performed by using the UniRef90 database [45]. Accordingly, we got the relative abundances of intestinal microbial taxonomic compositions, gene families, and metabolic pathways, respectively. In light of the compositionality nature of microbiome data [46], we conducted the centered log-ratio (CLR) transformation for raw relative abundance profiles with R package “zcomposition” prior to downstream differential abundance analyses.

### Co-evolution analysis based on shotgun metagenomic data of gut microbiota

Based on the correlation of the strain Lp082 with the indigenous microbes, we employed the MIDAS (Metagenomic Intra-Species Diversity Analysis System) pipeline to annotate mutations in the resident gut microbiota [47]. Briefly, we constructed a reference genome database that included 33 gut microbial genomes/species either with an average abundance more than 0.1% in real fecal samples or closely related to Lp082. Then we mapped the shotgun metagenomic sequencing reads from each microbiome to this reference database and quantified nucleotide variation along the entire genome. The samples at baseline in each host were set as the reference for the calculation of bacterial mutations occurred within hosts at other time points. The SNPs profiles for these intestinal microbes among the human and mouse models both in probiotic and placebo groups (Table S8 and Table S9).

### Experimental verification of SNPs and the phenotypic changes associated with SNPs

For SNPs verification, we retrieved the upstream and downstream 100-bp sequences of each SNP in the final set and designed primers (Table S2) for PCR amplification. The Sanger sequencing results of PCR products were used to verify the nucleotide status of SNPs acquired from the in silico analysis. After verification, 22 SNPs out of 71 putative SNPs were finally confirmed.

Further, we examined the potential phenotypic improvements of this strain caused by the selective single-nucleotide mutations in vitro, including the ability of rhamnose utilization, trehalose utilization (Fig. S2C), and acid tolerance. All of the mutants were isolated, stored in − 80 C freezer for 9 months before being used in the culture experiments. For the polysaccharides utilization experiments, the rhamnose/trehalose was set as the only source of carbohydrate in broth and the bacterial growth conditions including growth rate and the number of colonies were calculated. More specifically, after activation, 100 μL inoculum of Lp082 original strain and other mutants were put into sterilized lipid medium separately, incubating in a shaker with 120 r/min at 37 °C for 12 h. The absorbance at 600 nm was measured and recorded by a spectrophotometer after cultivating for 0 h, 2 h, 4 h, 6 h, 10 h, and 12 h. Rhamnose/trehalose carbon source limiting medium: peptone 10 g; yeast extract 5 g; beef extract 10 g; hydrogen diamine citrate 2 g; rhamnose/trehalose 20 g; MgSO_4_·7H_2_O 0.58 g; MnSO_4_·H_2_O 0.25 g; K_2_HPO_4_ 2 g; Na-acetate 5 g; Tween 80 1 mL; distilled water 1 L. For the acid tolerance experiment, the mutational strains and the ancient Lp082 strain were inoculated into the broth with pH values of 2, 2.5, 3.0, and 7.0 for 6 h respectively, and the strain survival rates were calculated.

### Statistical analysis

All statistical analyses were performed using R software (v3.5.1). PCoA analysis was performed in R using the ade4 package. CLR transformation was performed by the “zcomposition” package. The heatmap was constructed using the “pheatmap” package, and the evolutionary dynamics were built using the “ggmuller” package. The differential abundances of various profiles were tested with the Wilcoxon rank-sum test and were considered significantly different at *p* < 0.05. For boxplot construction, the package “ggpubr” was used. The co-occurrence network was calculated by the “SpiecEasi” correlations [25] and were visualized in Cytoscape (v3.7.1). For more details, please refer to the document deposited in Github:https://github.com/zhjch321123/Host-dependent-co-evolution-of-supplemented-probiotic.

## Supplementary Information

**Additional file 1: Figure S1**. The adaptive evolution of Lp082 under *in vitro* and *in vivo* conditions. (A) The genome sequence of the original Lp082 strains inoculated in the MRS broth at day 7, 14, 21 and 28. During the *in vitro* incubation period, no SNP was annotated in these strains. (B) The temporal pattern of sequencing coverage of two mobile elements inserted in Lp082 during its colonization in the gut of human and mouse. The x-axis represents the duration of probiotic colonization, while the y-axis represents the log10-transformed read coverage of each mobile element that can be inserted into the probiotic genome. Mobile Element 023 and 027 were identified in the plasmid 4 of this probiotic strain. (C-G) Phylogenetic trees constructed based on all Lp082 isolates in each time point. The number in tree branches represent the branch lengths among the isolates. **Figure S2**. The genes underwent *in vivo* evolution in the three hosts. (A) The heatmap shows the presence or absence of SNPs and mobile elements detected and experimentally verified in all isolates from human, mouse and zebrafish hosts. The probiotic isolates are shown in columns, while the verified SNPs or mobile elements corresponding to a probiotic isolate are shown in the rows. (B) The predictive protein structure of five genes underwent *in vivo* parallel evolution. (C) The phenotypic verification experiments of isolates related to trehalose utilization. No significant difference in trehalose utilization was found between the original probiotic strain and the mutational isolate. **Figure S3**. The alterations in the resident gut microbiome responding to the probiotic invasion in humans and mice. A, B) The boxplot indicating Bray-Curtis distance between samples in baseline and other time points both in the human and mouse model. The colored points represent stool samples collected at different time points. We found that there is a relatively large inter-individual variation in the gut microbiome between human hosts as compared to that in mice. (C) The heatmap shows intestinal species significantly changed responding to Lp082 ingestion in the mouse model. (D) The scatter plot shows the fold change of microbial metabolic pathways from Day 0 responding to Lp082 ingestion in the mouse model. However, no microbial species or functional contents in the human gut showed a significant association with probiotic ingestion potentially due to the large individuality observed in the human gut microbiome. **Figure S4**. The *in vivo* evolution of resident microbial strains in the gut under the selection of probiotic ingestion. (A) The number of SNPs occurred in the resident intestinal strains over the 28-days sampling period responding to probiotic ingestion in humans and mice. (B) Mantel tests quantifying the correlation between each pair of measurements (taxonomic profile, functional profile and SNP profile) on each sample collected from humans and mice. Each matrix showed results on the Mantel tests on a sample group (probiotic or placebo) from humans or mice. In each matrix, the values in the lower diagonal indicate the R values of the Mantel test, which range from -1 to 1, representing the correlation between a pair of measurements. The corresponding *p* values of the correlations are shown in the upper diagonal of the matrix. Results showed that probiotic ingestion resulted in a tighter coupling between SNP profiles and taxonomic or functional profiles in the gut of mice. (C) The relationship between the changes in the species-level Shannon diversity and the number of SNPs. The probiotic intake did lead to more fluctuations in the Shannon diversity than usual (placebo group) yet no significant correlation between change in Shannon diversity and mutation frequency was observed in both humans and mice.

**Additional file 2: Table S1**. The metadata of 109 Lp082 isolates (mutants). **Table S2**. The detailed summary of 71 SNPs computationally identified from Lp082 isolate genomes. **Table S3**. The Lp082 genes under the parallel evolution. **Table S4**. The Average Nucleotide Identity (ANI) profiling of 103 bacterial genomes (including Lp082) from *Lactiplantibacillus plantarum*. **Table S5**. Mobile elements related to Lp082 isolates. **Table S6**. The microbial taxonomic profiles of all fecal metagenome samples. **Table S7**. The microbial metabolic functional profiles of all fecal metagenome samples. **Table S8**. The number of SNPs annotated in the resident gut microbes. **Table S9**. The detailed summary of SNPs in the resident gut microbes that correlated with Lp082’s abundance.

## Data Availability

The sequence data reported in this paper have been deposited in the NCBI database (genome resequencing and metagenomic sequencing data: PRJNA594992, PRJNA597371, PRJNA590026, PRJNA597969 and PRJNA588621). All the project analysis code had been deposited in Github:https://github.com/zhjch321123/Host-dependent-co-evolution-of-supplemented-probiotic.
