# Supplementary material for: Candidate probiotic Lactiplantibacillus plantarum HNU082 rapidly and convergently evolves within human, mice, and zebrafish gut but differentially influences the resident microbiome
Source: Microbiome. 2021 Jun 30;9:151. doi: 10.1186/s40168-021-01102-0 (PMC8247228; doi:10.1186/s40168-021-01102-0)
Supplement: Supplementary file 2 — Additional file 1: Figure S1. The adaptive evolution of Lp082 under in vitro and in vivo conditions. (A) The genome sequence of the original Lp082 strains inoculated in the MRS broth at day 7, 14, 21 and 28. During the in vitro incubation period, no SNP was annotated in these strains. (B) The temporal pattern of sequencing coverage of two mobile elements inserted in Lp082 during its colonization in the gut of human and mouse. The x-axis represents the duration of probiotic colonization, while the y-axis represents the log10-transformed read coverage of each mobile element that can be inserted into the probiotic genome. Mobile Element 023 and 027 were identified in the plasmid 4 of this probiotic strain. (C-G) Phylogenetic trees constructed based on all Lp082 isolates in each time point. The number in tree branches represent the branch lengths among the isolates. Figure S2. The genes underwent in vivo evolution in the three hosts. (A) The heatmap shows the presence or absence of SNPs and mobile elements detected and experimentally verified in all isolates from human, mouse and zebrafish hosts. The probiotic isolates are shown in columns, while the verified SNPs or mobile elements corresponding to a probiotic isolate are shown in the rows. (B) The predictive protein structure of five genes underwent in vivo parallel evolution. (C) The phenotypic verification experiments of isolates related to trehalose utilization. No significant difference in trehalose utilization was found between the original probiotic strain and the mutational isolate. Figure S3. The alterations in the resident gut microbiome responding to the probiotic invasion in humans and mice. A, B) The boxplot indicating Bray-Curtis distance between samples in baseline and other time points both in the human and mouse model. The colored points represent stool samples collected at different time points. We found that there is a relatively large inter-individual variation in the gut microbiome between human ho [file 40168_2021_1102_MOESM2_ESM.pdf]

Candidate probiotic *Lactiplantibacillus plantarum* HNU082 rapidly and convergently evolves within human, mice, and zebrafish gut, but differently influence the resident microbiome

Shi Huang [2, 3#], Shuaiming Jiang [1#], Dongxue Huo [1#], Celeste Allaband[5], Mehrbod Estaki [2], Victor Cantu [6], Pedro Belda-Ferre [2, 3], Yoshiki Vázquez-Baeza [2, 3], Qiyun Zhu[2, 3], Chenchen Ma [1], Congfa Li [1,9], Amir Zarrinpar [3,7,8], Yang-Yu Liu [10], Rob Knight [2, 3, 4, 5 *], Jiachao Zhang[1, 2, 9 *]

[1] School of Food Science and Technology, Hainan University, Haikou, China

[2] UCSD Health Department of Pediatrics, University of California, San Diego, 9500 Gilman Drive, La Jolla, CA 92093, USA

[3] Center for Microbiome Innovation, Jacobs School of Engineering, University of California, San Diego, 9500 Gilman Drive, La Jolla, CA 92093, USA

[4] Department of Computer Science and Engineering, University of California, San Diego, 9500 Gilman Drive, La Jolla, CA 92093, USA

[5] Biomedical Sciences Graduate Program, University of California, San Diego, 9500 Gilman Drive, La Jolla, CA 92093, USA

[6] Department of Bioengineering, University of California, San Diego, 9500 Gilman Drive, La Jolla, CA 92093, USA

[7] UCSD Division of Gastroenterology, University of California, San Diego, 9500 Gilman Drive, La Jolla, CA 92093, USA

[8] VA San Diego Healthcare, 3350 La Jolla Village Dr, San Diego, CA 92161

[9] Key Laboratory of Food Nutrition and Functional Food of Hainan Province, Haikou 570228, China

[10] Channing Division of Network Medicine, Brigham and Women's Hospital and Harvard Medical School, Boston, Massachusetts, 02115, USA

[#] Contributed equally to this work

*Lead contact

Supplementary Figures

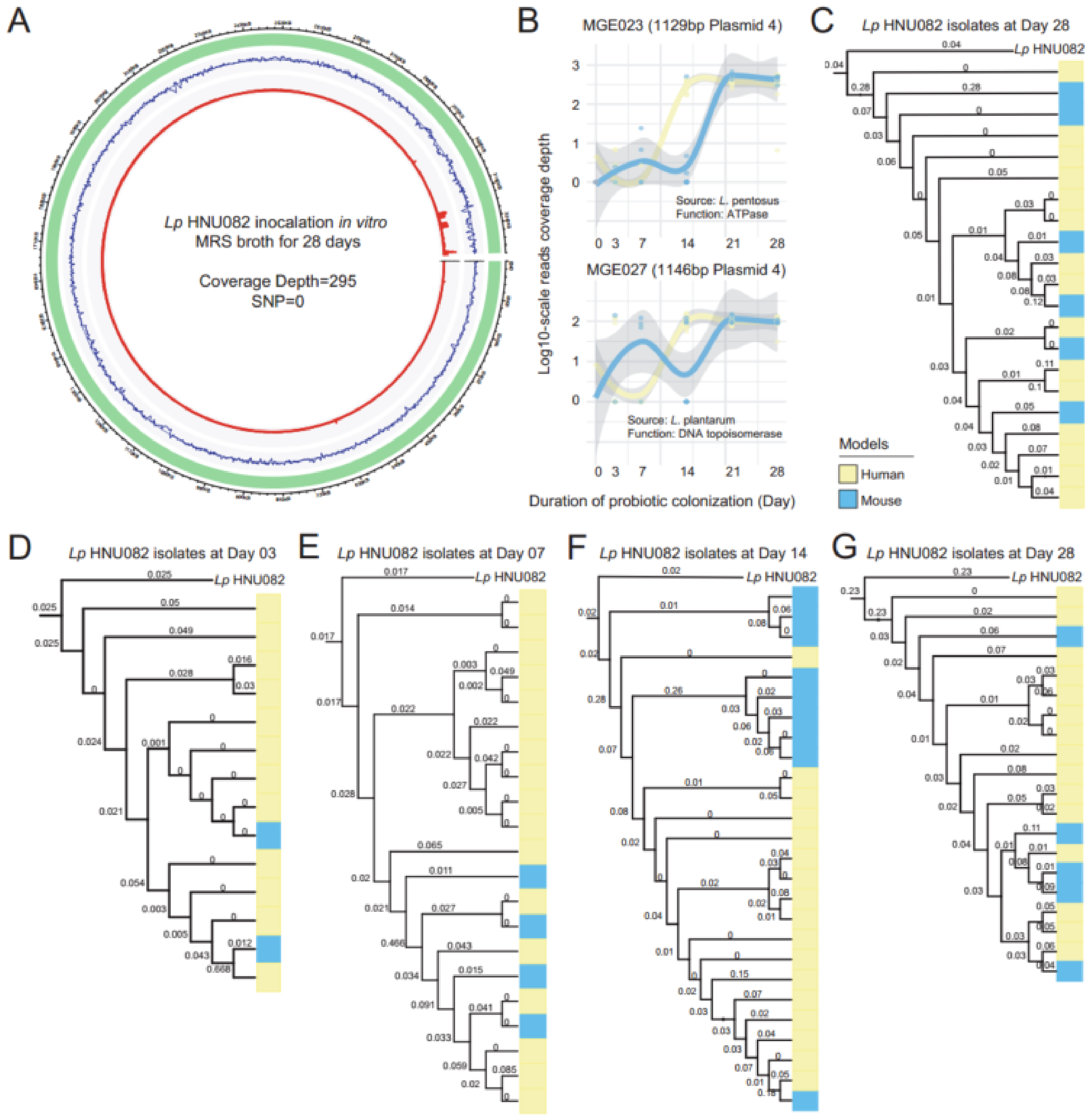

Figure S1. The adaptive evolution of Lp082 under *in vitro* and *in vivo* conditions. (**A**) The genome sequence of the original Lp082 strains inoculated in the MRS broth at day 7, 14, 21 and 28. During the *in vitro* incubation period, no SNP was annotated in these strains. (**B**) The temporal pattern of sequencing coverage of two mobile elements inserted in Lp082 during its colonization in the gut of human and mouse. The x-axis represents the duration of probiotic colonization, while the y-axis represents the log10-transformed read coverage of each mobile element that can be inserted into the probiotic genome. Mobile Element 023 and

027 were identified in the plasmid 4 of this probiotic strain. (**C-G**) Phylogenetic trees constructed based on all Lp082 isolates in each time point. The number in tree branches represent the branch lengths among the isolates.

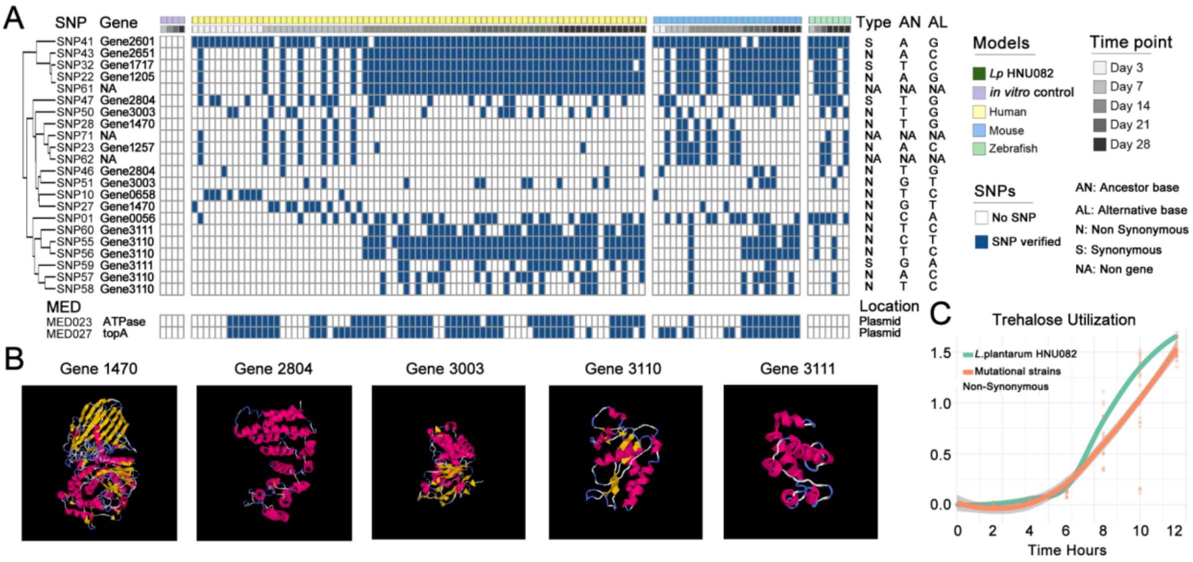

Figure S2. The genes underwent *in vivo* evolution in the three hosts. (**A**) The heatmap shows the presence or absence of SNPs and mobile elements detected and experimentally verified in all isolates from human, mouse and zebrafish hosts. The probiotic isolates are shown in columns, while the verified SNPs or mobile elements corresponding to a probiotic isolate are shown in the rows. (**B**) The predictive protein structure of five genes underwent *in vivo* parallel evolution. (**C**) The phenotypic verification experiments of isolates related to trehalose utilization. No significant difference in trehalose utilization was found between the original probiotic strain and the mutational isolate.

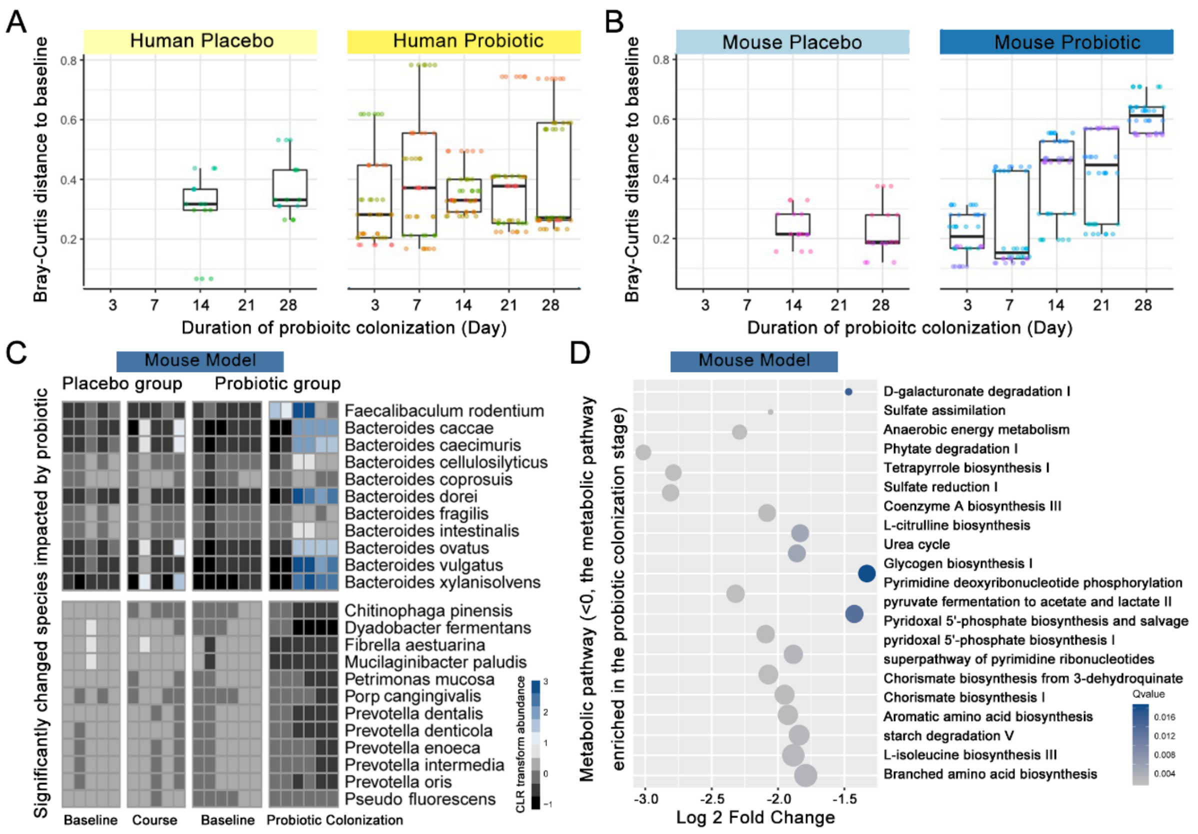

Figure S3. The alterations in the resident gut microbiome responding to the probiotic invasion in humans and mice. A, B) The boxplot indicating Bray-Curtis distance between samples in baseline and other time points both in the human and mouse model. The colored points represent stool samples collected at different time points. We found that there is a relatively large inter-individual variation in the gut microbiome between human hosts as compared to that in mice. (**C**) The heatmap shows intestinal species significantly changed responding to Lp082 ingestion in the mouse model. (**D**) The scatter plot shows the fold change of microbial metabolic pathways from Day 0 responding to Lp082 ingestion in the mouse model. However, no microbial species or functional contents in the human gut showed a significant association with probiotic ingestion potentially due to the large individuality observed in the human gut microbiome.

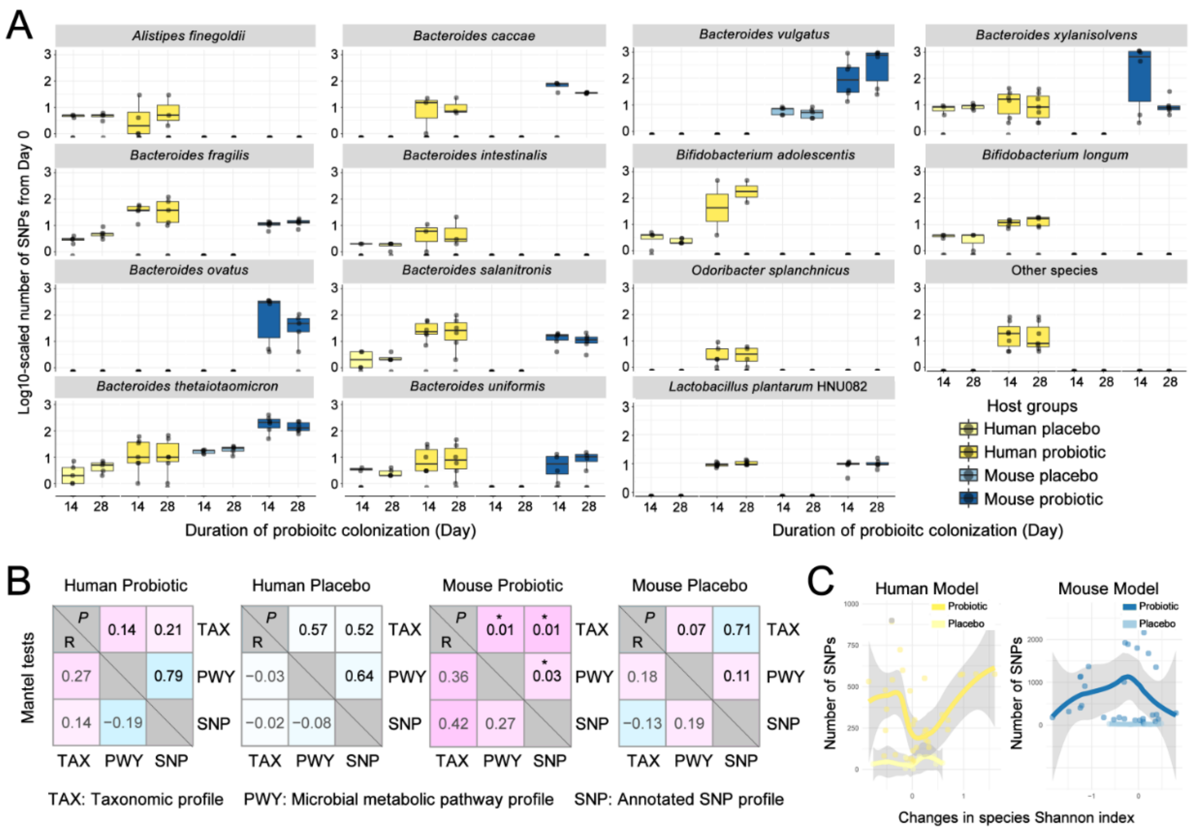

Figure S4. The *in vivo* evolution of resident microbial strains in the gut under the selection of probiotic ingestion. (**A**) The number of SNPs occurred in the resident intestinal strains over the 28-days sampling period responding to probiotic ingestion in humans and mice. (**B**) Mantel tests quantifying the correlation between each pair of measurements (taxonomic profile, functional profile and SNP profile) on each sample collected from humans and mice. Each matrix showed results on the Mantel tests on a sample group (probiotic or placebo) from humans or mice. In each matrix, the values in the lower diagonal indicate the R values of the Mantel test, which range from -1 to 1, representing the correlation between a pair of measurements. The corresponding *P* values of the correlations are shown in the upper diagonal of the matrix. Results showed that probiotic ingestion resulted in a tighter coupling between SNP profiles and taxonomic or functional profiles in the gut of mice. (**C**) The relationship between the changes in the species-level Shannon diversity and the number of SNPs. The probiotic intake did lead to more fluctuations in the Shannon diversity than

usual (placebo group) yet no significant correlation between change in Shannon diversity and mutation frequency was observed in both humans and mice.
